# Supplementary material for: A low-cost, open-source centrifuge adaptor for separating large volume clinical blood samples
Source: PLoS One. 2022 Jul 8;17(7):e0266769. doi: 10.1371/journal.pone.0266769 (PMC9269434; doi:10.1371/journal.pone.0266769)
Supplement: S1 File — (DOCX) [file pone.0266769.s001.docx]

Design, 3D-printing, and characterisation of a low-cost, open-source centrifuge adaptor for separating large volume clinical blood samples

Md Ehtashamul Haque, Linda Marriott, Noman Naeem, Taygan Henry, Alvaro J. Conde and Maïwenn Kersaudy-Kerhoas

**Supplementary Material**

**Supplementary Table S1:** Technical specification of SciSpin MINI Microfuge, model: SQ-6050

| **Rotor Capacity** | 8 x 1.5 / 2.2 ml or 16 x 0.2ml PCR |
| --- | --- |
| **Max Speed** | 7,000 RPM |
| **Max RCF** | 2,680g |
| **Timer** | Continuous operation |
| **Dimensions (DxWxH)** | 160mm x 170mm x 122mm |
| **Weight** | 0.5kg |
| **Noise level** | 45 dB |
| **Power** | 100-240V- /50Hz/60Hz 20W |
| **Rotor Capacity** | 8 x 1.5 / 2.2 ml or 16 x 0.2ml PCR |

**Supplementary Table S2:** Technical characteristics of Anycubic i3 Mega 3D printer

| **Technical Characteristics** | **Anycubic Mega I3 printer** |
| --- | --- |
| Printing technology | Fused Deposition Modelling |
| Standard nozzle | 0.4 mm |
| Printing bed size | 210 x 210 x 205 mm |
| Extruder head temperature | 275 °C (max) |
| Hot bed temperature | 100 °C (max) |
| Printing materials diameter | 1.75 mm |
| Printing material | PLA |

**Supplementary Table S3:** 3D printing setting for Ultimaker Cura 4.4

| **Technical Characteristics** | **Anycubic Mega I3 printer** |
| --- | --- |
| Printing technology | Fused Deposition Modelling |
| Standard nozzle | 0.4 mm |
| Printing bed size | 210 x 210 x 205 mm |
| Extruder head temperature | 275 °C (max) |
| Hot bed temperature | 100 °C (max) |
| Printing materials diameter | 1.75 mm |
| Printing material | PLA |

**Supplementary Table S4:** Motor power load of different designs at the loaded and unloaded condition

| **Design** | **Peak current (A)** | **Peak voltage (V)** | **Peak power (W)** | **Average current at full speed (A)** | **Average voltage at full speed (V)** | **Average power at full speed (W)** |
| --- | --- | --- | --- | --- | --- | --- |
| Design C unloaded | 1.54 | 17.9 | 20.3 | 0.7 | 17.8 | 11.6 |
| Design C loaded | 1.52 | 17.9 | 20.3 | 0.7 | 17.7 | 11.6 |
| Design D unloaded | 1.54 | 19.9 | 21.6 | 0.6 | 19.6 | 12.5 |
| Design D loaded | 1.51 | 19.9 | 20.7 | 0.6 | 19.8 | 12.6 |
| Design E0 unloaded | 1.43 | 22.2 | 20.5 | 0.6 | 22.1 | 13.8 |
| Design E0 loaded | 1.53 | 17.9 | 20.5 | 0.7 | 17.6 | 11.5 |

**Supplementary Table S5:** Design evaluation against requirements

|  | **A30** | **A37.5** | **A45** | **B30** | **B37.5** | **B45** | **C** | **D** | **E0** | **E2** | **E5** |
| --- | --- | --- | --- | --- | --- | --- | --- | --- | --- | --- | --- |
| **R1** | ✓ | ✓ | ✓ | ✓ | ✓ | ✓ | ✓ | ✓ | ✓ | ✓ | ✓ |
| **R2** | ✓ | ✓ | ✓ | ✓ | ✓ | ✓ | ✓ | ✓ | ✓ | ✓ | ✓ |
| **R3** | ✓ | ✓ | ✓ | ✓ | ✓ | ✓ | ✓ | ✓ | ✓ | ✓ | ✓ |
| **R4** | ✓ | ✓ | ✓ | ✓ | ✓ | ✓ | ✓ | ✓ | ✓ | ✓ | ✓ |
| **R5** | ✓ | ✓ | ✓ | ✓ | ✓ | ✓ | ✓ | ✓ | ✓ | ✓ | ✓ |
| **R6** | ✓ | ✓ | ✓ | ✓ | ✓ | ✓ | ✓ | ✓ | ✓ | ✓ | 🗶 |
| **R7** | 🗶 | 🗶 | 🗶 | 🗶 | 🗶 | 🗶 | 🗶 | 🗶 | 🗶 | ✓ | ✓ |
| **R8** | n/t | n/t | n/t | n/t | n/t | n/t | 🗶 | 🗶 | ✓ | ✓ | 🗶 |
| **R9** | ✓ | ✓ | ✓ | ✓ | ✓ | ✓ | ✓ | ✓ | ✓ | ✓ | ✓ |
| **R10** | 🗶 | 🗶 | 🗶 | 🗶 | 🗶 | 🗶 | ✓ | ✓ | ✓ | ✓ | ✓ |

**Supplementary Figure S1: Protocol for deflection measurements**


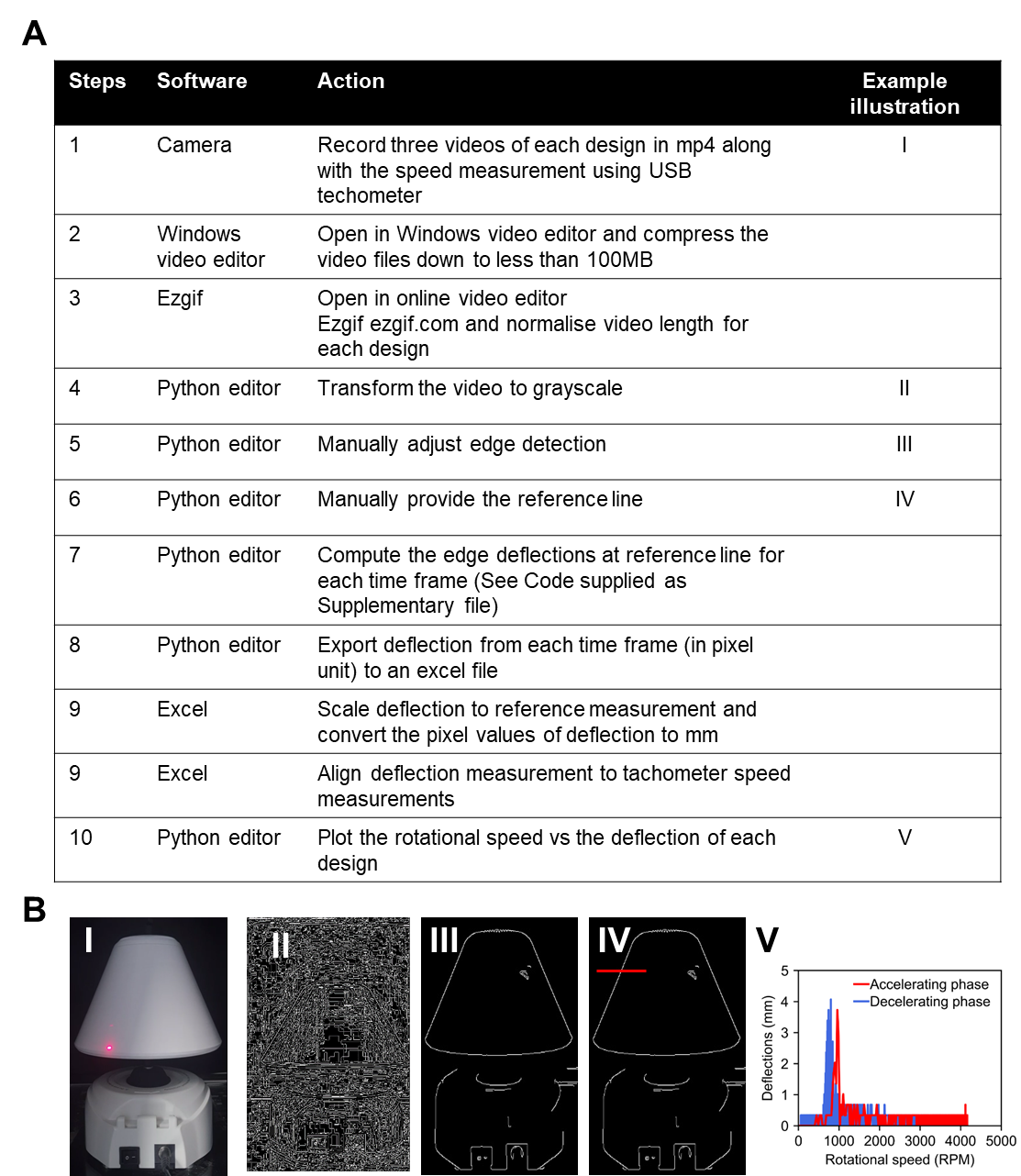


**Supplementary Figure S1:** **(A)** Step-by-step guide for the deflection measurements **(B)** Corresponding illustration for steps annotated I-V in (A). One video of design C has been uploaded as a separate Supplementary File as an example. All three video files for each design are available from FigShare https://doi.org/10.6084/m9.figshare.16762444.v1

**Supplementary Figure S2: Raw data for deflection measurements**


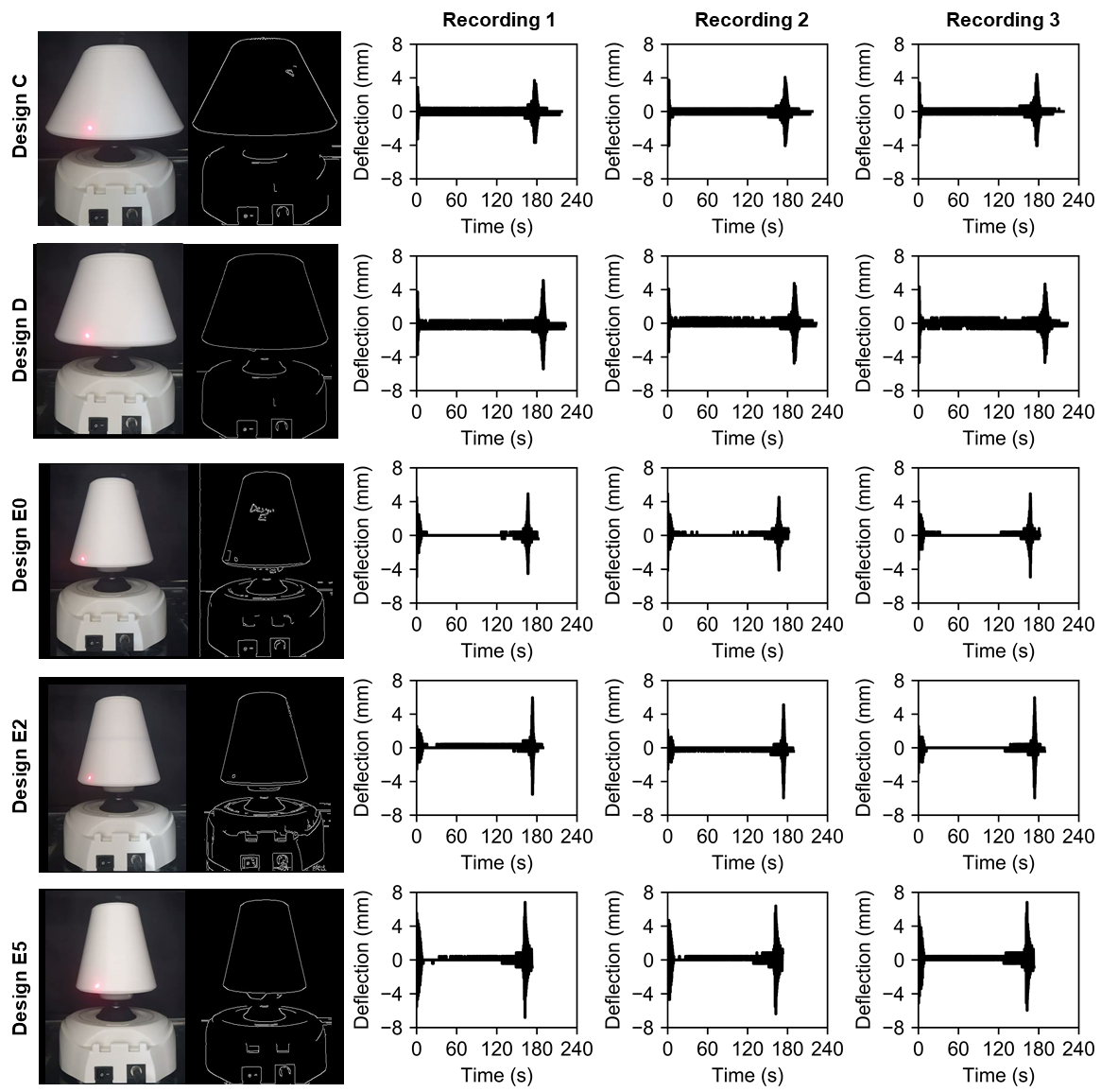


**Supplementary Figure S2:** Real-time deflection measurement from the recorded video of Designs C, D, E0, E2, E5

**Supplementary Figure S3: WBC count**


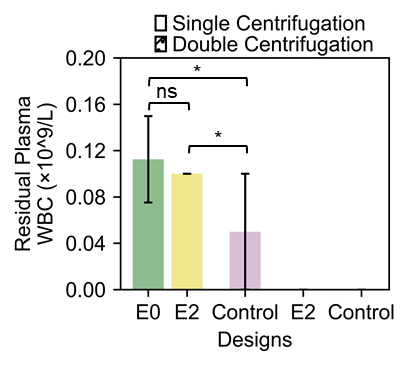


**Supplementary Figure S3**: WBC count on the residual plasma after single centrifugation and double centrifugation (A second centrifugation @12000 RCF, 10 minutes)

***S1 Code: Python code for deflection measurements. See separate file***

***S1 Video: Video recording of Design C. See separate file***
